# Temporal trends in the epidemiology of early‐onset colorectal cancer in England 2012–2021: Implications for early detection and diagnosis

**DOI:** 10.1111/codi.70660

**Published:** 2026-11-09

**Authors:** E. Haworth, L. Forster, E. Pennock, C. Dobson, L. Woods, L. Sharp

**Affiliations:** ^1^ Population Health Sciences Institute Newcastle University Newcastle‐upon‐Tyne UK; ^2^ London School of Hygiene & Tropical Medicine London UK; ^3^ NIHR Newcastle Patient Safety Research Collaboration Newcastle University Newcastle‐upon‐Tyne UK

**Keywords:** colorectal cancer, early‐onset, epidemiology

## Abstract

**Background:**

Early‐onset colorectal cancer (EOCRC; diagnosed <50 years) incidence is increasing globally; however, understanding of the epidemiology remains limited. We compared and contrasted EOCRC incidence patterns in England during 2012–2021 with late‐onset colorectal cancer (LOCRC; ≥50 years).

**Methods:**

All primary colorectal cancer (CRC) cases (351,900) were identified from the English National Cancer Registration Database. Percentages and age‐standardised incidence rates were calculated by socio‐demographic and geographical variables, tumour subsite, stage and route to diagnosis. Trends were assessed for 2012–2021, two quinquennia (2012–2016; 2017–2021) and COVID‐19 pandemic‐specific periods (1/1/2012–31/3/2020; 1/4/2020–31/12/2021). Incidence rate ratios (IRR) were derived using Poisson regression.

**Results:**

EOCRC accounted for 6% of CRC. Whilst more males were diagnosed with LOCRC (55.9%), EOCRC incidence varied little by sex. EOCRC incidence increased over time. The COVID‐19 pandemic was associated with a brief (~6 month) disruption in incidence trends for both EOCRC and LOCRC. For EOCRC, incidence of advanced stage disease rose markedly from 2012–2016 to 2017–2021 (IRR = 1.21 (1.16, 1.25)); incidence of both early and advanced‐stage LOCRC fell slightly over time (early‐stage IRR = 0.99 (0.98, 1.00); advanced‐stage IRR = 0.97 (0.96, 0.98)). A greater proportion of EOCRC (25.4%) were diagnosed as emergencies compared with LOCRC (20.2%). Regional variations were minimal for EOCRC but more pronounced for LOCRC.

**Conclusion:**

EOCRC incidence continues to rise and displays distinct epidemiological features compared to LOCRC. Higher rates of advanced disease, more frequent emergency diagnosis, and the sharpest increases in the youngest adults underscore growing challenges for timely detection and diagnosis.

**What does this paper add to the literature?**

Incidence of early‐onset CRC (EOCRC) continues to increase rapidly and displays distinct demographic and tumour characteristics compared to later‐onset disease; a higher frequency of emergency diagnoses and rising incidence of advanced disease in EOCRC highlight the importance of improved understanding of aetiology and barriers to early diagnosis in this population.

## INTRODUCTION

Incidence of early‐onset cancer—cancers diagnosed in adults under 50—has been increasing worldwide [1]. This rising burden has been observed across several cancers, including breast, endometrium, head and neck, pancreas, prostate, stomach, and colorectal cancer [1]. Early‐onset colorectal cancer (EOCRC) incidence was recently shown to be rising in 27 of the 50 countries examined in an international analysis of temporal trends [2]. Of these, England had the fourth greatest annual increase in EOCRC. However, contemporary data, including those detailing patterns and trends around the COVID‐19 pandemic, are sparse.

Colorectal cancer (CRC) is one of the most preventable cancers, with modifiable lifestyle factors playing a well‐established aetiological role [3]. In the UK, it is estimated that half of CRCs are due to lifestyle [4]. Work to date largely suggests that risk factors are similar across EOCRC and later‐onset CRC (LOCRC) [5]. Most CRCs develop from pre‐malignant polyps, either adenomas or serrated lesions [6]. Polyp detection and removal to prevent cancers (alongside earlier detection of cancers) provides the rationale for CRC screening. In England, screening consists of biennial faecal immunochemical testing (FIT) and, while the screening age‐range has recently been widened from 60–74 to 50–74, there are no plans to screen younger people. Moreover, only around 10% of CRCs in England are identified through screening: [7] the majority are diagnosed following presentation with symptoms [8]. Route to diagnosis (e.g. screening, urgent referral, emergency presentation) is associated with stage and, consequently survival [9]. Patterns in routes to diagnosis for EOCRC, and incidence by stage, in England currently remain unknown.

EOCRC is therefore a growing public health challenge, in England and beyond. While incidence trends have been previously described, these are primarily based on registry data up to the mid‐to‐late 2010s, with comparatively fewer analyses examining recent years [1, 2]. There is an urgent need to develop a fuller, contemporaneous understanding of the wider epidemiology of EOCRC, including geographic and socio‐economic distribution, routes to and stage at diagnosis, as well as how patterns changed (or not) during the health service disruptions of the COVID‐19 pandemic. This study investigated patterns of EOCRC incidence in England during 2012–2021 and compared these with LOCRC. Identifying similarities and differences in patterns across EOCRC and LOCRC could identify potential inequalities and areas of focus for interventions.

## METHODS

### Design

Retrospective, population‐based observational study using data from the English National Cancer Registration Dataset (NCRD).

### Data

#### National Cancer Registration Dataset (NCRD)

All newly incident primary malignant CRC tumours (defined using the International Classification of Diseases 10th revision (ICD‐10), codes C18‐C20) diagnosed in England from 1 January 2012 to 31 December 2021 were eligible for inclusion. These were identified by the National Cancer Registration and Analysis Service (NCRAS) within the NCRD. The NCRD is a population‐based cancer registry which includes data on all people diagnosed in England with malignant and pre‐malignant neoplasms since 1 January 1971 [10]. In patients with more than one registered tumour, the earliest diagnosis was included in the analysis (Figure S1).

### Clinical variables

We examined CRC overall along with four subsites: proximal colon (C18.0–C18.4), distal colon (C18.5–C18.7), other/unspecified colon (C18.8–C18.9) and rectum (C19–C20) [11].

Patients' age at diagnosis was provided in the dataset in 5‐year age bands. Tumours diagnosed in childhood or adolescence (before age 20) were excluded. EOCRC was defined as those remaining tumours diagnosed under 50 years of age [1, 12] and further stratified into three subgroups: 20–29 years, 30–39 years, and 40–49 years. LOCRC was defined as diagnoses occurring at age 50 years or older.

Stage at diagnosis was derived by NCRAS using patients' tumour (T), node (N), and metastases (M) information fields in the dataset, which are largely based on pathology reports [10]. Whilst pathology is not mandatory, cancer registration in England is, and the NHS aims to have at least 80% of cancer registry entries with full staging information [13]. In situ tumours (stage 0) were excluded [14]. Stage was grouped into “early” (stages I–II), “advanced” (stages III–IV), and “unknown”.

Route to diagnosis (RTD) was derived based on a previously published algorithm which tracks patient interactions with the healthcare system prior to a cancer diagnosis through several national, routine, health service datasets [8]. The variable was available for 2012–2020 and has eight categories: standard GP referral; urgent suspected cancer referral (USC); emergency presentation; other outpatient; screen‐detected; inpatient elective; death certificate only (DCO); and unknown. For this analysis, and following past work [15, 16], GP referral, other outpatient, and inpatient elective were categorised as “other primary care route” as all these routes will have commenced in primary care. Since both “DCO” (the very small number of cancer registrations based solely on death certificates) and “screening” (not offered for those under 50) routes had limited relevance to our primary research question, we categorised these two groups with tumours with an “unknown” route to diagnosis into the category “other”. This resulted in four groups for analysis: USC, other primary care, emergency presentation, and “other”.

### Demographic variables

Deprivation was assessed using quintiles of the Index of Multiple Deprivation 2019 (IMD), ranging from 1 (most deprived) to 5 (least deprived), based on the 2011 Lower‐Level Super Output Area (LSOA) corresponding to the patient's residential postcode at diagnosis [17]. Government region was also derived.

### Population denominators

Mid‐year population estimates by age group, sex, IMD, and government region were derived from the Office for National Statistics (ONS) data at LSOA level for the years 2012–2021 [18, 19]. Age‐standardised incidence rates were calculated using the European Standard Population 2013 (ESP) as the population standard.

### Statistical analyses

Percentages, age‐specific and directly age‐standardised incidence rates (ASIRs) per 100,000 were calculated for the study period as a whole and for two quinquennia: 2012–2016 and 2017–2021. Analyses of rates were stratified by sex, deprivation, region, subsite, stage at diagnosis, and RTD. Absolute rate differences were calculated, and Poisson regression was utilised to model the number of CRC diagnoses over time, using population estimates as the offset. Incidence rate ratios (IRR), CIs and *p*‐values have been reported. In supplementary analyses examining the impact of the COVID‐19 pandemic, two time periods were defined a priori: the pre‐pandemic period and pandemic period. Since the UK's first national lockdown began on 23 March 2020 [20], the end of March 2020 was chosen as the cutoff for the “pre‐pandemic period”. The “pandemic period” comprised of April 2020–December 2021.

In all figures, incidence rates were transformed to a log scale to aid visual comparison between EOCRC and LOCRC. All analyses were carried out in STATA version 18 [21].

## RESULTS

### Numbers and percentages of cases

A total of 351,900 people were diagnosed with CRC between 2012 and 2021 in England (Table 1). Of these, 94.0% (*n* = 330,665) were LOCRC and 6.0% (*n* = 21,235) EOCRC. The proportion of EOCRC was similar in 2012–2016 and 2017–2021 (6.0% vs. 6.1%).

**TABLE 1:** Number (with %s) of EOCRC and LOCRC diagnosed in England 2012–2021 by key variables.

Variable	Age 20–29		Age 30–39		Age 40–49		All EOCRC (age 20–49)		All LOCRC (age ≥ 50)	
	*n*	%	*n*	%	*n*	%	*n*	%	*n*	%
ALL PERSONS	1,459	6.9	6,047	28.5	13,729	64.7	21,235	100	330,665	100
Sex										
Male	685	3.2	2,978	14.0	7,131	33.6	10,794	50.8	184,707	55.9
Female	774	3.6	3,069	14.5	6,598	31.1	10,441	49.2	145,958	44.1
Diagnosis year										
2012	204	1.0	481	2.3	1,355	6.4	2,040	9.6	32,636	9.9
2013	188	0.9	500	2.4	1,309	6.2	1,997	9.4	31,785	9.6
2014	203	1.0	523	2.5	1,276	6.0	2,002	9.4	31,687	9.6
2015	192	0.9	571	2.7	1,296	6.1	2,059	9.7	32,388	9.8
2016	198	0.9	662	3.1	1,324	6.2	2,184	10.3	32,330	9.8
2017	112	0.5	634	3.0	1,293	6.1	2,039	9.6	32,348	9.8
2018	94	0.4	680	3.2	1,385	6.5	2,159	10.2	33,235	10.1
2019	92	0.4	711	3.3	1,439	6.8	2,242	10.6	34,659	10.5
2020	77	0.4	588	2.8	1,371	6.5	2,036	9.6	31,615	9.6
2021	99	0.5	696	3.3	1,681	7.9	2,476	11.7	37,982	11.5
IMD quintile										
1 (Most Deprived)	349	1.6	1,136	5.3	2,744	12.9	4,229	19.9	53,590	16.2
2	326	1.5	1,262	5.9	2,764	13.0	4,352	20.5	60,161	18.2
3	307	1.4	1,297	6.1	2,704	12.7	4,308	20.3	69,431	21.0
4	260	1.2	1,176	5.5	2,748	12.9	4,184	19.7	73,512	22.2
5 (Least Deprived)	217	1.0	1,176	5.5	2,769	13.0	4,162	19.6	73,971	22.4
Region										
East Midlands	112	0.5	406	1.9	1,203	5.7	1,721	8.1	29,705	9.0
East of England	168	0.8	741	3.5	1,500	7.1	2,409	11.3	38,616	11.7
London	297	1.4	1,225	5.8	2,043	9.6	3,565	16.8	32,811	9.9
North East	56	0.3	217	1.0	647	3.0	920	4.3	17,998	5.4
North West	169	0.8	655	3.1	1,864	8.8	2,688	12.7	46,122	13.9
South East	253	1.2	1,176	5.5	2,303	10.8	3,732	17.6	56,954	17.2
South West	128	0.6	624	2.9	1,419	6.7	2,171	10.2	39,404	11.9
West Midlands	144	0.7	518	2.4	1,431	6.7	2,093	9.9	36,412	11.0
Yorkshire and The Humber	132	0.6	485	2.3	1,319	6.2	1,936	9.1	32,643	9.9
Rural–Urban classification										
Rural Town	104	0.5	504	2.4	1,239	5.8	1,847	8.7	37,061	11.2
Rural Village	97	0.5	422	2.0	1,210	5.7	1,729	8.1	38,633	11.7
Urban Conurbation	633	3.0	2,696	12.7	6,005	28.3	9,334	44.0	148,771	45.0
Urban	626	2.9	2,425	11.4	5,275	24.8	8,326	39.2	106,200	32.1
Subsite										
Proximal Colon	798	3.8	2,108	9.9	3,791	17.9	6,697	31.5	120,479	36.4
Distal Colon	257	1.2	1,713	8.1	3,938	18.5	5,908	27.8	88,802	26.9
Other/Unspecified Colon	40	0.2	201	0.9	406	1.9	647	3.0	13,406	4.1
Rectum	364	1.7	2,025	9.5	5,594	26.3	7,983	37.6	107,978	32.7
Stage at diagnosis										
Early	736	3.5	1,925	9.1	4,517	21.3	7,178	33.8	134,299	40.6
Advanced	586	2.8	3,670	17.3	8,349	39.3	12,605	59.4	160,374	48.5
Unknown	137	0.6	452	2.1	863	4.1	1,452	6.8	35,992	10.9
RTD										
DCO	1	0.0	3	0.0	10	0.0	14	0.1	709	0.2
Emergency Presentation	764	3.6	1,679	7.9	2,953	13.9	5,396	25.4	66,885	20.2
GP Referral	278	1.3	1,601	7.5	3,286	15.5	5,165	24.3	60,678	18.4
Inpatient Elective	70	0.3	342	1.6	653	3.1	1,065	5.0	8,140	2.5
Other Outpatient	103	0.5	517	2.4	949	4.5	1,569	7.4	19,163	5.8
Screening	1	0.0	1	0.0	14	0.1	16	0.1	31,247	9.4
USC	87	0.4	927	4.4	3,603	17.0	4,617	21.7	99,622	30.1
Unknown*	155	0.7	977	4.6	2,261	10.6	3,393	16.0	44,221	13.4
Ethnicity										
Missing/unknown	131	0.6	388	1.8	889	4.2	1,408	6.6	18,755	5.7
Asian	111	0.5	398	1.9	798	3.8	1,307	6.2	6,357	1.9
Black	31	0.1	186	0.9	343	1.6	560	2.6	2,152	0.7
White	1,126	5.3	4,803	22.6	11142	52.5	17,071	80.4	29,9267	90.5
Other	44	0.2	180	0.8	398	1.9	622	2.9	3,164	1.0
Mixed	16	0.1	92	0.4	159	0.7	267	1.3	970	0.3

Abbreviations: DCO, Death certificate only; EOCRC, Early‐onset colorectal cancer; GP, General practitioner; IMD, Index of multiple deprivation; LOCRC, Late‐onset colorectal cancer; RTD, route to diagnosis; USC, Urgent suspected cancer.

* 73% of “unknown” route EOCRC cases and 86% of “unknown” LOCRC were diagnosed in 2021.

Across the study period, tumours in males accounted for 55.9% (*n* = 184,707) of LOCRCs compared to 50.8% (*n* = 10,794) of EOCRCs. Of EOCRC, 80.4% were diagnosed in people whose ethnicity was recorded as White, and 13.0% among people of Asian, Black, Mixed and Other ethnicities; figures for LOCRC were 90.5% and 3.8% (6.6% of EOCRC and 5.7% of LOCRC had unknown/missing ethnicity). There were more rectal cancers for EOCRC than LOCRC (37.6% vs. 32.7%), whilst a higher percentage of LOCRC were proximal colon tumours (36.4% vs. 31.5%). More EOCRCs had advanced stage disease at diagnosis than LOCRCs (stage III/IV: 59.4% compared to 48.5%). Unknown stage tumours for EOCRC and LOCRC were 6.8% and 10.9% respectively. The most frequent route to diagnosis for EOCRCs was emergency presentation (25.4%), followed by GP referral (24.3%) and USC referral (21.7%). In contrast, the largest percentage of LOCRCs were diagnosed through USC (30.1%), followed by emergency presentations (20.2%) and GP referral (18.4%).

### Incidence rates

#### Temporal trends

Age‐standardised incidence rates (ASIRs) for EOCRC were consistently lower than LOCRC (Figure 1). A significant 2% increase in EOCRC incidence was observed between 2012–2016 and 2017–2021 for both males (IRR: 1.02; *p* < 0.001) and females (IRR: 1.02; *p* < 0.001). In contrast, there was no meaningful evidence of temporal change in LOCRC incidence overall or when stratified by sex (Table S1).

**FIGURE 1:**
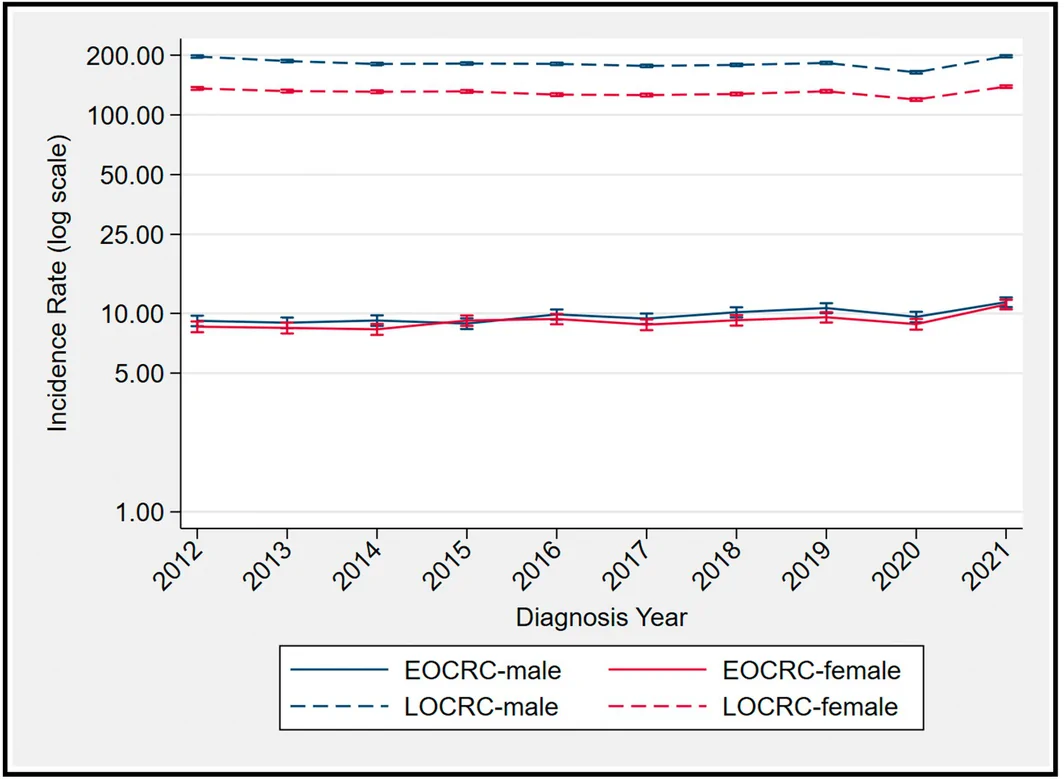
Age‐standardised incidence rates per 100,000, with 95% confidence intervals, for EOCRC and LOCRC by diagnosis year and sex, 2012–2021.

Across the pre‐pandemic period (2012–2020) ASIRs for EOCRC and LOCRC changed only gradually over time (Figure 2, Table S2). At the onset of the COVID‐19 pandemic (2020q2), a sharp disruption occurred, with both EOCRC and LOCRC incidence dropping markedly. This decline was followed by a subsequent rebound by late 2020. In 2021 EOCRC rates returned to their gradual upward trajectory, and LOCRC returned to the constant level observed previously.

**FIGURE 2:**
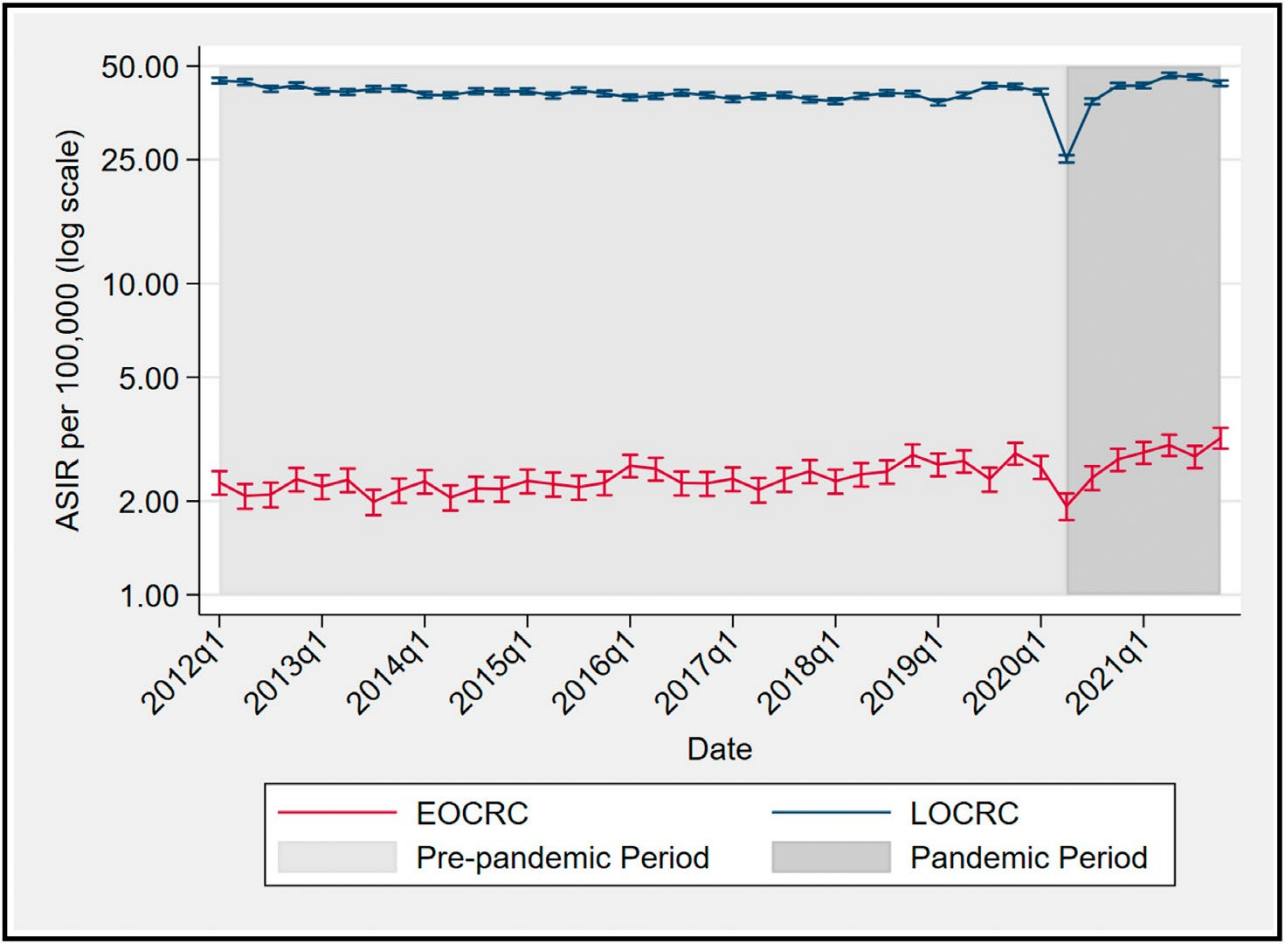
Quarterly age‐standardised incidence rates per 100,000, with 95% confidence intervals, for EOCRC and LOCRC by sex, 2012–2021.

#### Sex

Notable sex differences were observed across age groups (Table 2). Age‐standardised EOCRC incidence was higher in males (IRR: 1.05; 95% CI: 1.02, 1.08) overall. However, when stratified by age, males had slightly lower incidence in ages 20–29 (IRR: 0.85; 95% CI: 0.78, 0.92). There were no significant sex differences for ages 30–39 (IRR: 1.00; 95% CI: 0.96, 1.06). Males had higher incidence in ages 40–49 (IRR: 1.11; 95% CI: 1.07, 1.15). By contrast, sex differences were substantially larger in LOCRC, with males showing markedly higher incidence than females in ASIRs (IRR: 1.40; 95% CI: 1.39, 1.41).

**TABLE 2:** Age‐specific and age‐standardised incidence rates per 100,000 for EOCRC and LOCRC with 95% confidence intervals (CI) and M:F incidence rate ratios (IRR), 2012–2021.

	Incidence rate per 100,0000 (95% CI)		Absolute difference (95% CI)	M:F IRR (95% CI)	*p*‐Value
	Male	Female			
EOCRC (20–49)					
Age 20–29	1.8 (1.7, 1.9)	2.0 (1.9, 2.1)	−0.2 (−0.0, −0.0)	0.85 (0.78, 0.92)	<0.001*
Age 30–39	7.8 (7.5, 8.0)	7.8 (7.5, 8.0)	0.0 (−0.4, 0.4)	1.00 (0.96, 1.06)	0.837
Age 40–49	18.4 (18.0, 18.8)	16.6 (16.2, 17.0)	1.8 (1.2, 2.4)	1.11 (1.07, 1.15)	<0.001*
Age‐standardised	9.7 (9.5, 9.9)	9.1 (9.0, 9.3)	0.6 (0.3, 0.8)	1.05 (1.02, 1.08)	<0.001*
LOCRC (50+)					
Age‐standardised	182.4 (181.4, 183.2)	129.9 (129.2, 130.6)	52.5 (51.4, 53.6)	1.40 (1.39, 1.41)	<0.001*

Abbreviations: CI, Confidence interval; EOCRC, Early‐onset colorectal cancer; IRR, Incidence rate ratio; LOCRC, Late‐onset colorectal cancer.

* Statistical significance at the 5% level.

#### Subsite

Among males, patterns in incidence by subsite were similar for EOCRC and LOCRC. The highest rates were observed for the rectum, followed by proximal colon and then distal colon (Figure 3). Among females, a similar pattern was seen for EOCRC. However, for LOCRC, proximal colon cancer had the highest incidence, followed by rectum and distal colon. Between time periods, EOCRC incidence increased for rectal (males IRR: 1.12 (1.06, 1.19), females IRR: 1.18 (1.10, 1.26)) and distal colon (males IRR: 1.31 (1.21, 1.41), females IRR: 1.22 (1.14, 1.31)) cancers in both sexes—while declining for proximal (males IRR: 0.93 (0.87, 1.00), females IRR: 0.85 (0.79, 0.90)) and other/unspecified colon subsites (males IRR: 0.61 (0.49, 0.77), females IRR: 0.70 (0.56, 0.87)) (Table S3). In contrast, LOCRC incidence generally decreased across all subsites except for proximal colon, which increased (males IRR: 1.02 (1.01, 1.04), females IRR: 1.06 (1.05, 1.08)).

**FIGURE 3:**
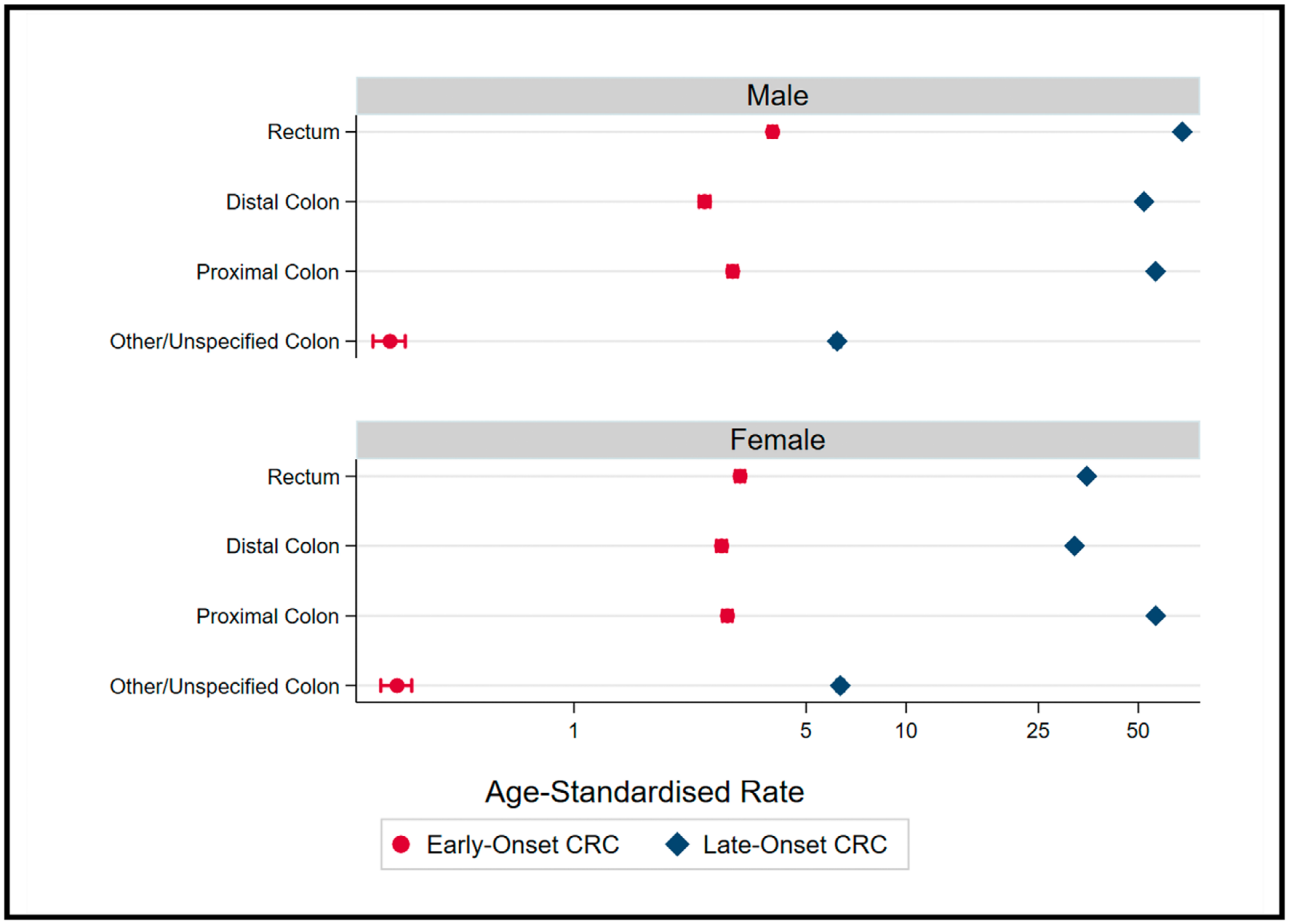
Age‐standardised incidence per 100,000 with 95% confidence intervals for EOCRC and LOCRC by sex and subsite, 2012–2021. 95% confidence intervals have been plotted but are not visible for all points due to their small magnitude.

#### Stage

Stage‐specific incidence trends revealed contrasting patterns between EOCRC and LOCRC (Table 3). Among EOCRC, incidence of early‐stage disease declined from 3.5 per 100,000 (95% CI: 3.4, 3.6) in 2012–2016 to 3.2 per 100,000 (95% CI: 3.1, 3.3) in 2017–2021 (IRR: 0.88; 95% CI: 0.84, 0.92; *p* < 0.001). Most notably, advanced‐stage EOCRC incidence rose from 5.3 per 100,000 (95% CI: 5.1, 5.4) in 2012–2016 to 6.5 per 100,000 (95% CI: 6.3, 6.7) in 2017–2021 (IRR: 1.21; 95% CI: 1.16, 1.25; *p* < 0.001). Cases with unknown stage had no statistically significant change over the same two time periods (IRR: 0.93; 95% CI: 0.84, 1.03; *p* = 0.174). By contrast, LOCRC showed small reductions in incidence rates across all stages.

**TABLE 3:** Age‐standardised incidence rates per 100,000 for EOCRC and LOCRC by stage and time period, absolute differences in ASIRs, and IRRs from Poisson regression comparing 2012–2016 to 2017–2021 (with 95% (CIs)).

Stage	Age standardised incidence rate per 100,000 (95% CI)		Absolute difference in incidence rates (95% CI)	IRR (95% CI)	*p*‐Value
	2012–2016	2017–2021			
Early					
EOCRC	3.5 (3.4, 3.6)	3.2 (3.1, 3.3)	−0.3 (−0.5, −0.2)	0.88 (0.84, 0.92)	<0.001*
LOCRC	67.1 (66.6, 67.6)	66.5 (65.9, 66.9)	−0.6 (−1.4, 0.1)	0.99 (0.98, 1.00)	0.030*
Advanced					
EOCRC	5.3 (5.1, 5.4)	6.5 (6.4, 6.7)	1.2 (1.0, 1.4)	1.21 (1.16, 1.25)	<0.001*
LOCRC	80.9 (80.3, 81.5)	78.6 (78.0, 79.1)	−2.4 (−3.1, −1.6)	0.97 (0.96, 0.98)	<0.001*
Unknown					
EOCRC	0.7 (0.6, 0.7)	0.7 (0.6, 0.7)	0.0 (−0.1, 0.0)	0.93 (0.84, 1.03)	0.174
LOCRC	18.5 (18.2, 18.7)	17.4 (17.1, 17.6)	−1.1 (−1.5, −0.7)	0.94 (0.92, 0.96)	<0.001*

*Note*: *p*‐Values correspond to Poisson regressions run to compare ASIRs between 2012–2016 and 2017–2021.

Abbreviations: CI, Confidence interval; EOCRC, Early‐onset colorectal cancer; IRR, Incidence rate ratio; IRR, incidence rate ratio; LOCRC, Late‐onset colorectal cancer.

* Statistical significance at the 5% level.

#### Routes to diagnosis

Across both EOCRC and LOCRC, “other primary care routes” accounted for the largest proportion of diagnoses (36.7% and 26.6% respectively) (Figure 4). Notable differences were observed in other pathways: EOCRC cases were more frequently diagnosed via emergency presentation than LOCRC (25.4% vs. 20.2%), whereas LOCRC cases were more commonly diagnosed following USC referral (Figure 4). Between time periods, routes to diagnosis shifted significantly for both EOCRC and LOCRC (All *p* < 0.001; Table S4). Emergency presentations declined in both groups, more sharply for EOCRC than LOCRC. Diagnoses via other primary care routes rose for EOCRC and LOCRC. USC referrals increased slightly for EOCRC but fell for LOCRC. As expected, “other” routes (which includes screening) accounted for a meaningful proportion of LOCRC but significantly less EOCRC cases (Figure 4).

**FIGURE 4:**
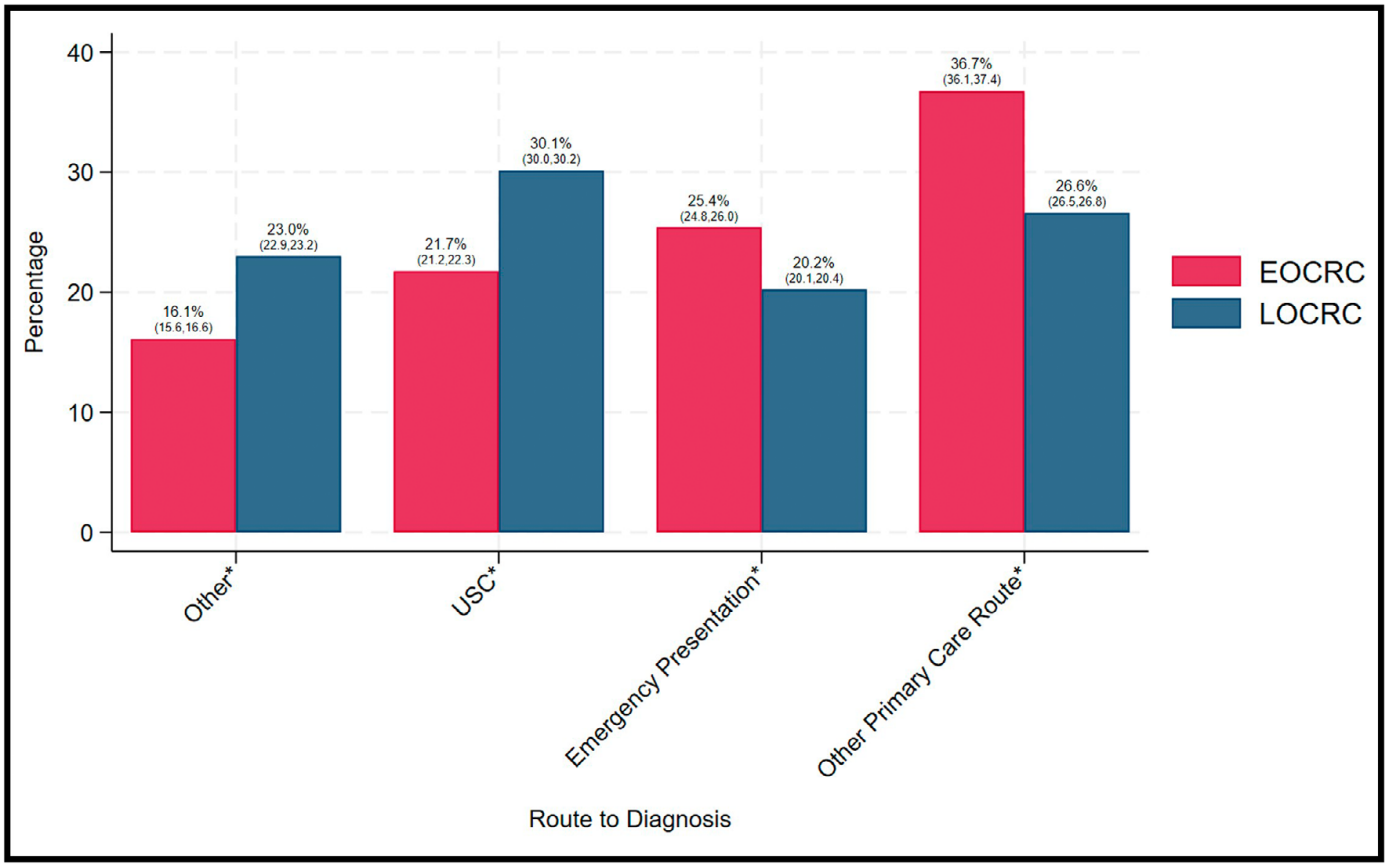
Percentage and 95% CIs of EOCRC and LOCRC by Route to Diagnosis, 2012–2020. CI, Confidence Interval; EOCRC, Early‐onset Colorectal Cancer; LOCRC, Late onset Colorectal Cancer; USC, urgent suspected cancer referral; “Other Primary Care Route” is comprised of GP referrals, other outpatient and inpatient elective routes, and “Other” is comprised of cancers diagnosed via “unknown” route, screening or from death certificates only (DCO). *Indicates the 95% CIs for EOCRC and LOCRC do not overlap, providing evidence of a difference for that referral route.

#### Deprivation

There was no evidence of a statistically significant deprivation gradient for men for either EOCRC or LOCRC, although there was some evidence that women living in more deprived areas had slightly lower incidence than those living in affluent areas (Figure S2).

#### Region

The highest EOCRC incidence among males was observed in the South East (ASIR: 11.0 per 100,000; 95% CI: 10.5, 11.5), and the lowest was in London (9.6; 95% CI: 9.2, 10.8) (Figure 5, Supplementary Table 6). For females, London also had the lowest incidence (8.7; 95% CI: 8.3, 9.1) while the South West had the highest (10.8; 95% CI: 10.1, 11.4). LOCRC showed greater variability across regions: male incidence was highest in the North East (212.7; 95% CI: 208.6, 216.8) and lowest in London (162.5; 95% CI: 160.1, 164.9), whereas female rates were lowest in London (119.3; 95% CI: 117.3, 121.2) and highest in the South West (147.7; 95% CI: 145.6, 149.9).

**FIGURE 5:**
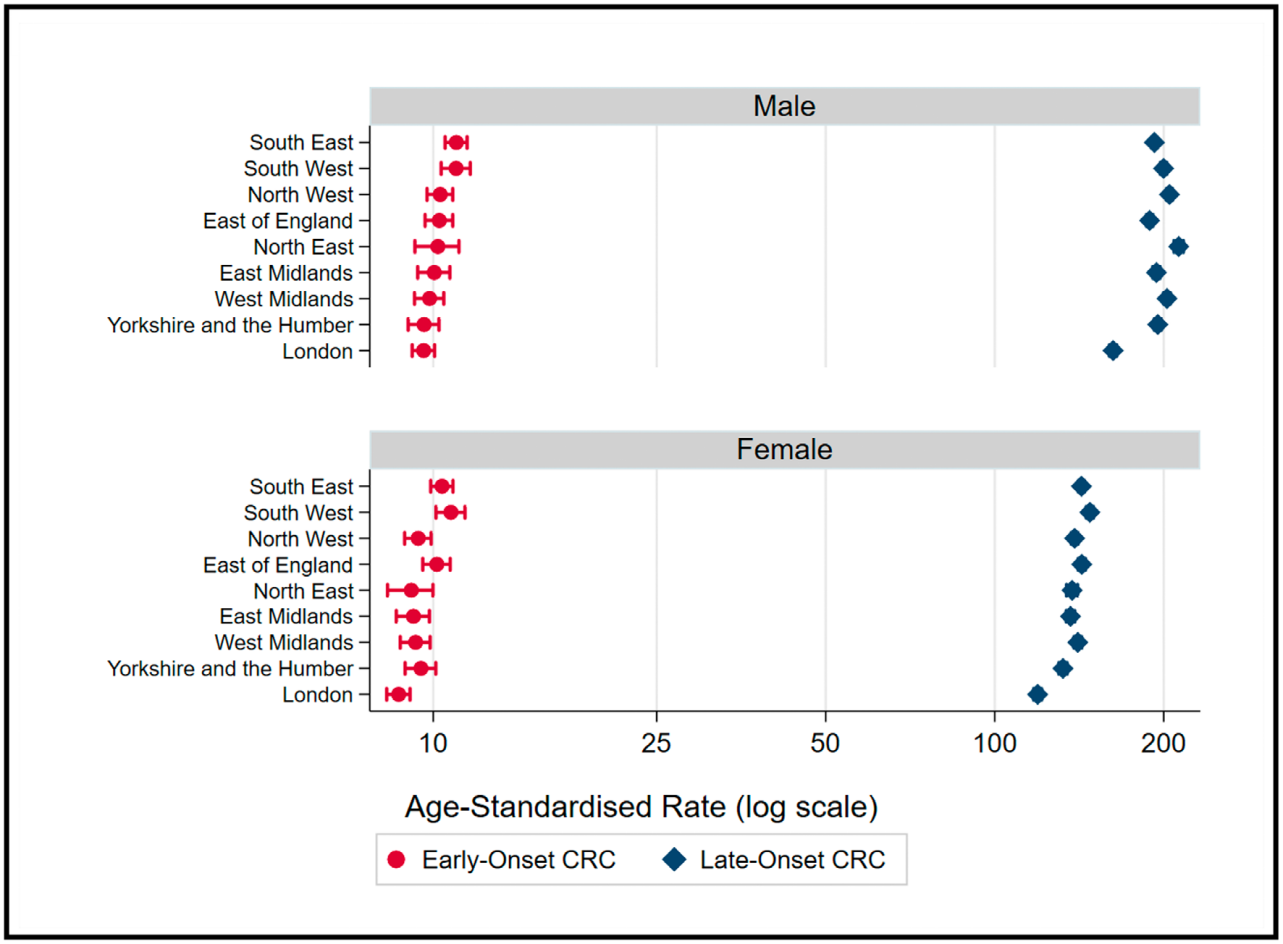
Age‐standardised incidence rate per 100,000 for EOCRC and LOCRC by sex and region. 95% confidence intervals have been plotted but are not visible for all points due to their small magnitude.

Between the two time periods, the largest significant increases in EOCRC incidence were observed in the South West (males IRR: 1.21 (1.07, 1.36), females IRR: 1.19 (1.05, 1.34)) and London (males IRR: 1.14 (1.04, 1.25), females IRR: 1.13 (1.03, 1.24)) (Table 4). Most regions showed moderate but statistically insignificant EOCRC increases for both sexes. In contrast, LOCRC rates were largely stable or declined slightly between the two time periods, particularly among males in London (IRR: 0.92 (0.90, 0.96)), the North West (IRR: 0.97 (0.94, 0.99)), the South East (IRR: 0.97 (0.95, 0.99)), the South West (IRR: 0.95 (0.93, 0.98)), and Yorkshire and The Humber (IRR: 0.97 (0.94, 1.00)). Female LOCRC trends showed decreases in the East of England (IRR: 0.96 (0.93, 0.99)), London (IRR: 0.97 (0.93, 1.00)), the South East (IRR: 0.95 (0.93, 0.98)), and the South West (IRR: 0.96 (0.93, 0.98)).

**TABLE 4:** Age‐standardised incidence rates per 100,000 of EOCRC and LOCRC by sex, region and period, with 95% confidence intervals (CI), absolute differences in ASIRs and IRRs from Poisson regression comparing 2012–2016 to 2017–2021 (95% CIs).

Region	Age‐standardised incidence rate per 100,000 (95% CI)		Absolute difference	IRR (95% CI)	*p*‐Value
	2012–2016	2017–2021	In incidence rates (95% CI)		
East Midlands					
EOCRC					
Male	9.2 (8.3, 10.1)	10.9 (9.9, 11.9)	1.7 (0.4, 3.0)	1.13 (0.99, 1.29)	0.061
Female	9.1 (8.2, 10.0)	9.4 (8.5, 10.3)	0.3 (−0.6, 1.2)	1.00 (0.88, 1.15)	0.970
LOCRC					
Male	195.7 (191.7, 200.0)	192.7 (188.7, 196.7)	−3.0 (−7.3, 1.3)	0.99 (0.96, 1.02)	0.359
Female	134.7 (131.4, 138.1)	138.0 (134.7, 141.3)	3.3 (−0.4, 7.0)	1.03 (0.99, 1.06)	0.154
East of England					
EOCRC					
Male	9.9 (9.1, 10.7)	10.6 (9.7, 11.4)	0.7 (−0.2, 1.6)	1.05 (0.94, 1.18)	0.359
Female	9.5 (8.8, 10.3)	10.8 (10.0, 11.6)	1.3 (0.2, 2.3)	1.13 (1.01, 1.27)	0.029*
LOCRC					
Male	189.5 (185.8, 193.2)	189.1 (184.6, 191.7)	−0.3 (−3.9, 3.2)	0.99 (0.97, 1.02)	0.673
Female	146.3 (143.2, 149.4)	139.7 (136.9, 142.6)	−6.6 (−10.9, −2.3)	0.96 (0.93, 0.99)	0.003*
London					
EOCRC					
Male	8.9 (8.3, 9.5)	10.3 (10.7, 11.0)	1.4 (−5.0, 7.8)	1.14 (1.04, 1.25)	0.006*
Female	8.12 (7.6, 8.8)	9.1 (8.5, 9.7)	1.0 (0.2, 1.7)	1.13 (1.03, 1.24)	0.011*
LOCRC					
Male	167.7 (164.2, 171.2)	157.8 (154.6, 161.0)	−9.9 (−15.6, −4.1)	0.92 (0.90, 0.96)	<0.001*
Female	121.1 (118.4, 124.0)	117.6 (114.9, 120.2)	−3.6 (−6.7, −0.5)	0.97 (0.93, 1.00)	0.032*
North East					
EOCRC					
Male	10.1 (8.8, 11.3)	10.3 (9.0, 11.7)	0.3 (−1.1, 1.6)	0.95 (0.80, 1.14)	0.607
Female	8.9 (7.7, 10.0)	9.5 (8.2, 10.7)	0.6 (−0.7, 1.9)	1.00 (0.83, 1.21)	0.960
LOCRC					
Male	212.1 (206.2, 218.0)	213.3 (207.6, 219.0)	1.2 (−4.5, 6.9)	1.01 (0.97, 1.05)	0.700
Female	134.6 (130.2, 139.0)	139.9 (135.6, 144.3)	5.3 (0.2, 10.4)	1.04 (0.99, 1.09)	0.101
North West					
EOCRC					
Male	10.2 (9.5, 11.0)	10.4 (9.6, 11.2)	0.2 (−0.6, 1.0)	0.97 (0.88, 1.08)	0.606
Female	9.1 (8.4, 9.9)	9.7 (8.9, 10.4)	0.5 (−0.3, 1.3)	1.02 (0.91, 1.14)	0.729
LOCRC					
Male	208.6 (205.0, 212.2)	201.2 (197.8, 204.6)	−7.4 (−12.4, −2.5)	0.97 (0.94, 0.99)	0.008*
Female	139.2 (136.4, 142.0)	138.3 (135.67, 141.1)	−0.9 (−3.6, 1.9)	0.99 (0.97, 1.02)	0.702
South East					
EOCRC					
Male	10.4 (9.7, 11.1)	11.7 (10.9, 12.4)	1.3 (0.3, 2.2)	1.10 (1.01, 1.21)	0.035*
Female	10.0 (9.33, 10.7)	10.7 (10.1, 11.4)	0.8 (0.0, 1.5)	1.07 (0.98, 1.17)	0.143
LOCRC					
Male	195.8 (192.7, 198.9)	189.4 (186.5, 192.4)	−6.4 (−10.7, −2.1)	0.97 (0.95, 0.99)	0.004*
Female	146.5 (144.0, 149.1)	139.2 (136.8, 141.5)	−7.4 (−11.7, −3.0)	0.95 (0.93, 0.98)	<0.001*
South West					
EOCRC					
Male	9.8 (9.0, 10.7)	12.2 (11.3, 13.2)	2.4 (0.9, 3.9)	1.21 (1.07, 1.36)	0.002*
Female	9.7 (8.9, 10.6)	11.8 (10.9, 12.8)	2.1 (0.7, 3.5)	1.19 (1.05, 1.34)	0.005*
LOCRC					
Male	205.1 (201.1, 209.0)	195.1 (191.5, 198.8)	−9.9 (−16.0, −3.9)	0.95 (0.93, 0.98)	0.001*
Female	151.5 (148.3, 154.6)	144.3 (141.3, 147.2)	−7.2 (−11.8, −2.6)	0.96 (0.93, 0.98)	0.002*
West Midlands					
EOCRC					
Male	9.6 (8.8, 10.4)	10.1 (9.3, 11.0)	0.5 (−0.4, 1.4)	1.00 (0.89, 1.13)	0.944
Female	9.6 (8.8, 10.5)	9.0 (8.2, 9.8)	−0.6 (−1.5, 0.2)	0.89 (0.79, 1.01)	0.069
LOCRC					
Male	204.5 (200.5, 208.5)	201.0 (197.2, 204.8)	−3.5 (−7.7, 0.6)	0.99 (0.96, 1, 02)	0.394
Female	141.6 (138.4, 144.8)	139.7 (136.6, 142.7)	−1.9 (−5.1, 1.3)	0.99 (0.96, 1.02)	0.526
Yorkshire and The Humber					
EOCRC					
Male	9.4 (8.5, 10.2)	9.9 (9.0, 10.8)	0.5 (−0.4, 1.4)	1.00 (0.94, 1.13)	0.983
Female	9.12 (8.4, 10.0)	9.8 (9.0, 10.7)	0.7 (−0.3, 1.6)	1.02 (0.90, 1.16)	0.707
LOCRC					
Male	198.1 (194.1, 202.2)	192.5 (188.6, 196.3)	−5.7 (−10.4, −0.9)	0.97 (0.94, 1.00)	0.049*
Female	131.3 (128.1, 134.4)	133.3 (130.2, 136.4)	2.0 (−1.2, 5.2)	1.01 (0.98, 1.05)	0.408

*Note*: *p*‐Values correspond to Poisson regressions to compare changes in ASIRs between 2012–2016 and 2017–2021.

Abbreviations: CI, Confidence interval; EOCRC, Early‐onset colorectal cancer; IRR, Incidence rate ratio; LOCRC, Late‐onset colorectal cancer.

* Statistical significance at the 5% level.

## DISCUSSION

### Key findings

This study examined the epidemiology of EOCRC in comparison to LOCRC in England between 2012 and 2021. Four key findings were identified.

Firstly, EOCRC rates increased during the period 2012–2021, in contrast with LOCRC incidence rates which were stable. The upward trend in EOCRC was similar in men and women. For both EOCRC and LOCRC, the COVID‐19 pandemic was associated with only a short‐term disruption to the incidence trend, which returned to pre‐pandemic levels within 6 months of the first national lock‐down in March 2020.

Secondly, a clear temporal shift toward more advanced‐stage disease at diagnosis among EOCRC cases, compared to LOCRC, was demonstrated. Thirdly, and likely a reflection of findings for stage, EOCRC were more commonly diagnosed via emergency routes than LOCRC.

Finally, while LOCRC showed pronounced regional variation, these patterns were not mirrored in EOCRC: incidence of EOCRC rose over time in all regions and did not vary by deprivation.

### Comparison to existing studies

The rising incidence of EOCRC presented here is consistent with international evidence of sustained increases across multiple high‐income countries [2, 22]. In this study—the most contemporary analysis for England to date—we have shown the rising trend persists into the 2020s. Studies from the United States, Canada, Australia, and Europe have all reported similar trends [23]. Increasing exposure to carcinogenic risk factors in younger cohorts has been suggested to elevate lifetime CRC risk and a cohort effect has been observed [23, 24]. This is broadly compatible with our observation of fastest rising incidence in the youngest age group. Several aetiological explanations for the cohort effect have been proposed including: increased consumption of ultra‐processed foods [25, 26]; rising childhood and adolescent obesity [27]; reduced physical activity [28]; increased exposure to carcinogenic chemicals [29]; and environmentally‐induced gut microbiome and metabolic health changes during critical developmental periods [26, 27, 28]. Understanding which exposures are driving the cohort effect is crucial to guide targeted interventions to reduce EOCRC incidence.

More advanced‐stage disease at diagnosis for EOCRC patients, and rising incidence of advanced disease over time, are concerning, because of the relationship between stage and survival [30]. The stage‐specific incidence rates we present provide an absolute measure of the population burden of disease at each stage (rather than the relative proportion of cases within each stage). This therefore shows that EOCRC patients have (sometimes many) more potential years of life lost (YLL) when diagnosed with advanced disease than LOCRC [31]. In addition to the personal impact on patients and their families, premature deaths from cancer also have a significant economic impact on society [32]. Although a previous US study, of data from 1998 to 2011, reported that patients <50 were more frequently diagnosed with regional or distant disease than older patients [33], as far as we are aware, the current study is the first to note a larger rise in incidence of advanced than early disease in EOCRC.

Some data suggests that EOCRCs develop differently, and more aggressively, than LOCRCs [33, 34], which may explain our findings regarding stage. Younger patients may also experience longer diagnostic pathways [31, 35], with misappraisal and misattribution of EOCRC symptoms resulting in referral delays [36, 37]. The overlap between symptoms of cancer and common gynaecological conditions in young women may also contribute to diagnostic delay [36]. Lengthy primary care intervals, driven by lower clinical suspicion and diagnostic wandering [21, 22] could also explain the observed higher prevalence of emergency presentations among EOCRC patients. Although CRC screening begins at 45 in some countries (e.g. USA, Australia) [38, 39], this remains contentious in the UK, due to the high cost and low yield under 50 [40]. It is imperative, therefore, that understanding of EOCRC diagnostic pathways in England is improved, and any modifiable individual, practitioner, and system level factors that may be impacting timeliness of diagnosis identified.

### Strengths and limitations

A major strength of this study is its use of contemporary, population‐level data, enabling robust assessment of recent trends in EOCRC and LOCRC incidence, stage distribution, and diagnostic routes. Moreover, the NCRD follows internationally agreed standards for registration and coding [41], which enhance the reliability of incidence and staging data. The large sample size maximises statistical power for subgroup analyses, which have identified subtle but important differences in the epidemiology of EOCRC and LOCRC; such patterns may not be visible in smaller or single‐centre, non‐population‐based datasets because of small numbers and/or selection bias.

The study period captures a critical transitional era in colorectal cancer diagnostics, preceding the adoption of faecal immunochemical testing into symptomatic pathways [42], but capturing the COVID‐19 pandemic, which substantially impacted healthcare utilisation, screening and diagnostic services. The findings provide a valuable baseline against which to measure future evaluations of diagnostic pathway changes.

In terms of limitations, the observational design prohibits causal inference regarding drivers of EOCRC incidence. Moreover, certain variables may be poorly reported. For example, we observed that a substantially higher proportion of EOCRC than LOCRC occurred in people from non‐white ethnic groups (13.0% vs. 3.8%)—a potentially very important finding. However, it is well recognised that routine NHS ethnicity data varies in quality, consistency and completeness [43], making it difficult to draw firm conclusions from these data.  Crude comparison with available census data [44] suggests that the ethnic distribution of cases is broadly similar to the ethnic composition of the underlying population. However, since more detailed ethnic breakdown of census data by age, sex and ethnic group are unavailable, ethnicity‐specific incidence rates could not be calculated. Consequently, our data on ethnicity should be interpreted with caution due to the much higher levels of ethnic diversity in younger populations [44].

Finally, screen‐detected, DCO and unknown routes to diagnosis were combined into a single ‘other’ category. Although screen‐detected and DCO represent distinct and clinically meaningful pathways, very few EOCRC cases were identified through these routes in this cohort; this is entirely as expected given invitations to the Bowel Cancer Screening Programme commence at age 50. Therefore, these categories were grouped to mitigate issues arising from sparse data.

## CONCLUSIONS

In England EOCRC incidence continues to rise. Distinctive and important epidemiological characteristics are evident, including higher (and rising) rates of advanced‐stage disease and more frequent diagnoses through emergency routes. Further research into aetiological factors and diagnostic pathways is urgently required to inform primary and secondary prevention initiatives.

## AUTHOR CONTRIBUTIONS

**E. Haworth:** Data curation; formal analysis; writing – original draft; writing – review and editing; methodology. **L. Forster:** Methodology; data curation; formal analysis; visualization; writing – original draft; writing – review and editing. **E. Pennock:** Data curation; formal analysis; writing – review and editing; visualization; methodology. **C. Dobson:** Conceptualization; methodology; funding acquisition; supervision; writing – review and editing. **L. Woods:** Conceptualization; methodology; supervision; funding acquisition; writing – review and editing. **L. Sharp:** Conceptualization; methodology; writing – review and editing; supervision; funding acquisition.

## FUNDING INFORMATION

This work was funded by a grant from Health Data Research UK North. LS is supported by the National Institute for Health and Care Research (NIHR) Newcastle Patient Safety Research Collaboration (PSRC) [grant number: NIHR204291]. The views expressed are those of the authors and not necessarily those of the NIHR or the Department of Health and Social Care. The funders of the study had no role in the study design, the collection, analysis and interpretation of data, and writing of the report, or the decision to submit the article for publication.

## CONFLICT OF INTEREST STATEMENT

The authors declare that they have no conflicts of interest.

## ETHICS STATEMENT

The programme of work of which this study is part obtained a favourable ethical opinion from the Proportionate Review Sub‐committee of the West Midlands‐Edgbaston Research Ethics Committee on October 16th 2019 (REF19/WM/0317).

## Supporting information


**Figure S1:** Summary of steps taken in data preparation.
**Figure S2:** IRR and CIs comparing IMD quintiles for EOCRC and LOCRC.
**Table S1:** IRRs derived from Poisson regression models fitted to examine the temporal trend in incidence comparing 2012–2016 with 2017–2021 (95% CIs).
**Table S2:** Quarterly age‐standardised incidence rates per 100,000 (with 95% confidence intervals) for EOCRC and LOCRC over the pre‐pandemic and pandemic periods.
**Table S3:** Absolute change in incidence of EOCRC and LOCRC and IRRs (with 95% confidence intervals and *p* values) from Poisson regression between 2012–2016 and 2017–2021, by sex and subsite.
**Table S4:** Absolute and percentage changes in proportion of EOCRC and LOCRC between 2012–2016 and 2017–2020 by diagnostic pathway.
**Table S6:** Age‐standardised incidence rates per 100,000 for EOCRC and LOCRC from 2012 to 2021 by sex and region.

## Data Availability

Research data are not shared.
