# Supplementary material for: Temporal trends in the epidemiology of early‐onset colorectal cancer in England 2012–2021: Implications for early detection and diagnosis
Source: Colorectal Dis. 2026 Nov 9;28(11):e70660. doi: 10.1111/codi.70660 (PMC13748430; doi:10.1111/codi.70660)
Supplement: Supplementary file 1 [file CODI-28-0-s001.docx]

Supplementary Material

Supplementary Figure 1: Summary of steps taken in data preparation

Supplementary Table 1: IRRs derived from Poisson regression models fitted to examine the temporal trend in incidence comparing 2012-2016 with 2017-2021 (95% CIs)

|  |  | IRR (95 % CI) |
| --- | --- | --- |
| Male | EOCRC | 1.02 (1.01,1.02) |
|  | LOCRC | 1.00 (0.99,1.00) |
| Female | EOCRC | 1.02 (1.01,1.02) |
|  | LOCRC | 1.00 (1.00,1.00) |
| Sex X Diagnosis year interaction | EOCRC | 1.00 (0.99,1.01) |
|  | LOCRC | 1.00 (1.00,1.00) |

Abbreviations: EOCRC = Early onset colorectal cancer; LOCRC = Late onset colorectal cancer; IRR = Incidence rate ratio; CI = Confidence Interval

Footnote: p-values have not been reported to avoid potentially misleading interpretations of statistical significance arising from the large sample size.

Footnote: The first two rows represent models fitted separately for age-standardised annual rates of EOCRC and LOCRC incidence for males and females. The third row represents a model which included both males and females and fitted an interaction term to evaluate differences in the temporal trend between the sexes.

Supplementary Table 2: Quarterly age-standardised incidence rates per 100,000 (with 95% confidence intervals) for EOCRC and LOCRC over the pre-pandemic and pandemic periods

| Covid-19 Period | | Age-standardised incidence rates (95% CI) | |
| --- | --- | --- | --- |
|  |  | EOCRC | LOCRC |
| Pre-pandemic | 2012q1 | 2.3 (2.1,2.5) | 44.9 (43.9,45.9) |
|  | 2012q2 | 2.1 (1.9,2.3) | 44.4 (43.5,45.4) |
|  | 2012q3 | 2.1 (1.9,2.3) | 42.2 (41.3,43.2) |
|  | 2012q4 | 2.4 (2.2,2.6) | 43.3 (42.4,44.3) |
|  | 2013q1 | 2.2 (2.0,2.4) | 41.6 (40.7,42.4) |
|  | 2013q2 | 2.3 (2.1,2.5) | 41.3 (40.4,42.2) |
|  | 2013q3 | 1.2 (1.8,2.2) | 42.2 (41.3,43.2) |
|  | 2013q4 | 2.2 (2.0,2.4) | 42.4 (41.4,43.3) |
|  | 2014q1 | 2.3 (2.1,2.5) | 40.4 (39.5,41.3) |
|  | 2014q2 | 2.1 (1.9,2.2) | 40.3 (39.4,41.2) |
|  | 2014q3 | 2.2 (2.0,2.4) | 41.6 (40.7,42.5) |
|  | 2014q4 | 2.2 (2.0,2.4) | 41.4 (40.5,42.3) |
|  | 2015q1 | 2.3 (2.1,2.5) | 41.5 (40.6,42.4) |
|  | 2015q2 | 2.3 (2.1,2.5) | 40.2 (39.3,41.1) |
|  | 2015q3 | 2.2 (2.0,2.4) | 41.7 (40.8,42.6) |
|  | 2015q4 | 2.3 (2.1,2.5) | 40.8 (40.9,41.7) |
|  | 2016q1 | 2.6 (2.4,2.8) | 39.7 (38.8,40.5) |
|  | 2016q2 | 2.5 (2.3,2.8) | 40.1 (39.2,41.0) |
|  | 2016q3 | 2.3 (2.1,2.5) | 41.0 (40.2,41.9) |
|  | 2016q4 | 2.3 (2.1,2.5) | 40.3 (39.4,41.2) |
|  | 2017q1 | 2.4 (2.2,2.6) | 39.1 (38.3,40.0) |
|  | 2017q2 | 2.2 (2.0,2.4) | 40.1 (39.2,40.9) |
|  | 2017q3 | 2.4 (2.1,2.6) | 40.3 (39.5,41.2) |
|  | 2017q4 | 2.5 (2.3,2.7) | 39.0 (38.2,39.9) |
|  | 2018q1 | 2.3 (2.1,2.5) | 38.6 (37.7,39.4) |
|  | 2018q2 | 2.4 (2.2,2.7) | 40.1 (39.3,41.0) |
|  | 2018q3 | 2.5 (2.3,2.7) | 41.0 (40.1,41.9) |
|  | 2018q4 | 2.8 (2.6,3.0) | 40.7 (39.9,41.6) |
|  | 2019q1 | 2.6 (2.4,2.8) | 38.2 (37.4,39.0) |
|  | 2019q2 | 2.7 (2.5,2.9) | 40.3 (39.4,41.1) |
|  | 2019q3 | 2.4 (2.1,2.6) | 43.2 (42.3,44.1) |
|  | 2019q4 | 2.9 (2.6,3.1) | 43.0 (42.1,43.8) |
|  | 2020q1 | 2.6 (2.4,2.8) | 41.5 (40.6,42.3) |
| Pandemic | 2020q2 | 1.9 (1.7,2.1) | 25.2 (24.5,25.9) |
|  | 2020q3 | 2.4 (2.2,2.6) | 38.5 (37.7,39.3) |
|  | 2020q4 | 2.7 (2.5,3.0) | 43.3 (42.4,44.1) |
|  | 2021q1 | 2.9 (2.7,3.1) | 43.3 (42.4,44.2) |
|  | 2021q2 | 3.0 (2.7,3.3) | 46.7 (45.7,47.6) |
|  | 2021q3 | 2.8 (2.6,3.0) | 46.1 (45.1,47.0) |
|  | 2021q4 | 3.2 (3.0,3.4) | 44.1 (43.2,45.0) |

Abbreviations: EOCRC = Early onset colorectal cancer; LOCRC = Late onset colorectal cancer; CI = Confidence interval

|  | Site | Sex | Absolute change in incidence rates from 2012-16 to 2017-21 (95% CI) | IRR (95% CI) | P-value |
| --- | --- | --- | --- | --- | --- |
| EOCRC | Rectum | Male | 0.6  (0.3,0.8) | 1.12 (1.06,1.19) | <0.001* |
|  |  | Female | 0.6  (0.4,0.8) | 1.18 (1.10,1.26) | <0.001* |
|  | Distal Colon | Male | 0.7  (0.5,0.9) | 1.31 (1.21,1.41) | <0.001* |
|  |  | Female | 0.6  (0.4,0.8) | 1.22 (1.14,1.31) | <0.001* |
|  | Proximal Colon | Male | -0.1  (-0.3,0.1) | 0.93 (0.87,1.00) | 0.039* |
|  |  | Female | -0.4  (-0.6,-0.2) | 0.85 (0.79,0.90) | <0.001* |
|  | Other/Unspecified Colon | Male | -0.1  (-0.2,-0.1) | 0.61 (0.49,0.77) | <0.001* |
|  |  | Female | -0.1  (-0.2,-0.0) | 0.70 (0.56,0.87) | 0.001* |
| LOCRC | Rectum | Male | -3.5  (-4.5,-2.5) | 0.95 (0.94,0.96) | <0.001* |
|  |  | Female | -3.3  (-4.0,-2.6) | 0.91 (0.89,0.93) | <0.001* |
|  | Distal Colon | Male | -1.0  (-1.9,-0.1) | 0.98 (0.96,1.00) | 0.022* |
|  |  | Female | -0.9  (-1.5,-0.2) | 0.97 (0.95,0.99) | 0.009* |
|  | Proximal Colon | Male | 1.4  (0.5,2.3) | 1.02 (1.01,1.04) | 0.003* |
|  |  | Female | 3.4  (2.5,4.3) | 1.06 (1.05,1.08) | <0.001* |
|  | Other/Unspecified Colon | Male | -2.0  (-2.3,-1.7) | 0.72 (0.69,0.76) | <0.001* |
|  |  | Female | -1.8  (-2.1,-1.5) | 0.76 (0.72,0.79) | <0.001* |

Supplementary Table 3: Absolute change in incidence of EOCRC and LOCRC and IRRs (with 95% confidence intervals and p values) from Poisson regression between 2012-2016 and 2017-2021, by sex and subsite

Abbreviations: EOCRC = Early onset colorectal cancer; LOCRC = Late onset colorectal cancer; CI = Confidence interval

Footnote: p-values correspond to Poisson regressions run to compare ASIRs between 2012-2016 and 2017-2021, and * indicates statistical significance at the 5% level

Supplementary Table 4: Absolute and percentage changes in proportion of EOCRC and LOCRC between 2012-2016 and 2017-2020 by diagnostic pathway

| Route to Diagnosis | | Percentage | | Absolute change from 2012-16 to 2017-21 | % Change | P-value |
| --- | --- | --- | --- | --- | --- | --- |
|  |  | 2012-2016 | 2017-2020 |  |  |  |
| Emergency Presentation | EOCRC | 31.3 | 19.9 | -11.4 | -36.4 | <0.001* |
|  | LOCRC | 23.2 | 17.4 | -5.8 | -24.8 | <0.001* |
| USC | EOCRC | 20.6 | 22.9 | 2.3 | 11.1 | <0.001* |
|  | LOCRC | 31.6 | 28.7 | -2.9 | -9.1 | <0.001* |
| Other primary care routes | EOCRC | 48.1 | 57.1 | 9 | 18.8 | <0.001* |
|  | LOCRC | 34.8 | 44.9 | 10.2 | 29.2 | <0.001* |
| Other Routes | EOCRC | 0.1 | 0.2 | 0.1 | 54.6 | <0.001* |
|  | LOCRC | 10.5 | 8.9 | -1.5 | -14.1 | <0.001* |

Abbreviations: EOCRC = Early onset colorectal cancer; LOCRC = Late onset colorectal cancer

*Footnote: “Other primary care routes” comprises of GP referral, other outpatient and inpatient elective and “Other routes” comprises of screening, DCO and unknown routes. P-values were derived from Pearson’s χ^2^tests comparing the percentage of patients diagnosed via each route between each time period, conducted separately for EOCRC and LOCRC.*

Supplementary Figure 2: IRR and CIs comparing IMD quintiles for EOCRC and LOCRC

Abbreviations: EOCRC = Early onset colorectal cancer; LOCRC = Late onset colorectal cancer; IRR = incidence rate ratio; CI = confidence interval

Supplementary Table 6: Age-standardised incidence rates per 100,000 for EOCRC and LOCRC from 2012-2021 by sex and region

| Region | | | Age-Standardised incidence rate per 100,000 (95% CI) |  |
| --- | --- | --- | --- | --- |
|  |  |  |  |  |
| East Midlands | EOCRC | Male | 10.0 (9.4, 10.7) |  |
|  |  | Female | 9.2 (8.6,9.9) |  |
|  | LOCRC | Male | 194.1 (191.2,197.1) |  |
|  |  | Female | 136.4 (134.1,138.8) |  |
| East of England | EOCRC | Male | 10.3 (9.7,10.8) |  |
|  |  | Female | 10.2 (9.6,10.7) |  |
|  | LOCRC | Male | 188.8 (186.2,191.3) |  |
|  |  | Female | 142.9 (140.8,145.0) |  |
| London | EOCRC | Male | 9.6 (9.2,10.8) |  |
|  |  | Female | 8.7 (8.3,9.1) |  |
|  | LOCRC | Male | 162.5 (160.1,164.9) |  |
|  |  | Female | 119.3 (117.3,121.1) |  |
| North East | EOCRC | Male | 10.2 (9.3,11.1) |  |
|  |  | Female | 9.1 (8.3,10.0) |  |
|  | LOCRC | Male | 212.7 (208.6,216.8) |  |
|  |  | Female | 137.3 (134.2,140.4) |  |
| North West | EOCRC | Male | 10.3 (9.8,10.8) |  |
|  |  | Female | 9.4 (8.9,9.9) |  |
|  | LOCRC | Male | 204.8 (202.3,207.2) |  |
|  |  | Female | 138.8 (136.8,140.7) |  |
| South East | EOCRC | Male | 11.0 (10.5,11.5) |  |
|  |  | Female | 10.4 (9.9,10.8) |  |
|  | LOCRC | Male | 192.5 (190.3,1.9) |  |
|  |  | Female | 142.7 (140.9,144.4) |  |
| South West | EOCRC | Male | 11.0 (10.3,11.6) |  |
|  |  | Female | 10.8 (10.1,11.4) |  |
|  | LOCRC | Male | 199.9 (197.2,202.5) |  |
|  |  | Female | 147.7 (145.6,149.) |  |
| West Midlands | EOCRC | Male | 9.9 (9.3,10.4) |  |
|  |  | Female | 9.3 (8.7,9.9) |  |
|  | LOCRC | Male | 202.7 (199.9,205.) |  |
|  |  | Female | 140.6 (138.9,142.8) |  |
| Yorkshire and The Humber | EOCRC | Male | 9.6 (9.0,10.2) |  |
|  |  | Female | 9.5 (8.9,10.1) |  |
|  | LOCRC | Male | 195.2 (192.4,198.0) |  |
|  |  | Female | 132.3 (130.1,134.5) |  |

Abbreviations: EOCRC = Early onset colorectal cancer; LOCRC = Late onset colorectal cancer; CI = Confidence interval
